# Implications of ACC/AHA Versus ESC/EAS LDL-C Recommendations for Residual Risk Reduction in ASCVD: A Simulation Study From DA VINCI

**DOI:** 10.1007/s10557-022-07343-x

**Published:** 2022-05-14

**Authors:** Antonio J. Vallejo-Vaz, Sarah Bray, Guillermo Villa, Julia Brandts, Gaia Kiru, Jennifer Murphy, Maciej Banach, Stefano De Servi, Dan Gaita, Ioanna Gouni-Berthold, G. Kees Hovingh, Jacek J. Jozwiak, J. Wouter Jukema, Robert Gabor Kiss, Serge Kownator, Helle K. Iversen, Vincent Maher, Luis Masana, Alexander Parkhomenko, André Peeters, Piers Clifford, Katarina Raslova, Peter Siostrzonek, Stefano Romeo, Dimitrios Tousoulis, Charalambos Vlachopoulos, Michal Vrablik, Alberico L. Catapano, Neil R. Poulter, Kausik K. Ray, On behalf of the DA VINCI Study Investigators

**Affiliations:** 1 School of Public Health, Imperial College London, London, UK; 2 Department of Medicine, Faculty of Medicine, University of Seville, Seville, Spain; 3 Clinical Epidemiology and Vascular Risk, Instituto de Biomedicina de Sevilla, IBiS/Hospital Universitario Virgen del Rocío/Universidad de Sevilla/CSIC, Seville, Spain; 4 Global Biostatistical Science, Amgen Ltd, Cambridge, UK; 5 Global Health Economics, Amgen Europe (GmbH), Rotkreuz, Switzerland; 6 Imperial Centre for Cardiovascular Disease Prevention Imperial Clinical Trials Unit, Department of Primary Care and Public Health, School of Public Health, Imperial College London, Stadium House, 68 Wood Lane, London, W12 7RH UK; 7 Department of Internal Medicine I, University Hospital RWTH Aachen, Aachen, Germany; 8 Imperial Clinical Trials Unit, Imperial College London, London, UK; 9 Department of Hypertension, Medical University of Łódź, Łódź, Poland; 10 Polish Mother’s Memorial Hospital–Research Institute (PMMHRI), Łódź, Poland; 11 Cardiovascular Research Centre, University of Zielona Góra, Zielona Góra, Poland; 12 IRCCS MultiMedica, Milan, Italy; 13 Institutul de Boli Cardiovasculare, Fundatia Cardioprevent, Victor Babeş University of Medicine and Pharmacy, Timisoara, Romania; 14 Centre for Endocrinology, Diabetes and Preventive Medicine, University of Cologne, Faculty of Medicine and University Hospital Cologne, Cologne, Germany; 15 Department of Vascular Medicine, Amsterdam UMC, University of Amsterdam, Meibergdreef 9, Amsterdam, Netherlands; 16 Department of Family Medicine and Public Health, Faculty of Medicine, University of Opole, Opole, Poland; 17 Leiden University Medical Center, Leiden, Netherlands; 18 Hungarian Army Medical Center, Budapest, Hungary; 19 Centre Cardiologique et Vasculaire, Thionville, France; 20 Department of Neurology, Stroke Centre Rigshospitalet, Copenhagen, Rigshospitalet Denmark; 21 Faculty of Health and Medical Sciences, University of Copenhagen, Copenhagen, Denmark; 22 Trinity College Dublin, Dublin, Ireland; 23 Advanced Lipid Management and Research Centre, Tallaght University Hospital, Dublin, Ireland; 24 Universitat Rovira I Virgili, IISPV, CIBERDEM, Saint Joan University Hospital, Reus, Spain; 25 Emergency Cardiology Department, Institute of Cardiology, Kiev, Ukraine; 26 Cliniques Universitaires Saint Luc, Brussels, Belgium; 27 Imperial Hospitals NHS Trust (Hammersmith Campus), London, UK; 28 Slovak Medical University, Bratislava, Slovakia; 29 Krankenhaus Barmherzige Schwestern Linz, Linz, Austria; 30 Department of Molecular and Clinical Medicine, University of Gothenburg, Gothenburg, Sweden; 31 Clinical Nutrition, Department of Medical and Surgical Sciences, Magna Graecia University, Catanzaro, Italy; 32 Cardiology Department, Sahlgrenska University Hospital, Gothenburg, Sweden; 33 National and Kapodistrian University of Athens, Medical School, Athens, Greece; 34 1st Medical Faculty, Charles University and General University Hospital, Prague, Czech Republic

**Keywords:** Atherosclerotic cardiovascular disease, LDL-C, Lipid-lowering, Statins, Cardiovascular risk, Cardiovascular disease prevention

## Abstract

**Purpose:**

Low-density lipoprotein cholesterol (LDL-C) recommendations differ between the 2018 American College of Cardiology/American Heart Association (ACC/AHA) and 2019 European Society of Cardiology/European Atherosclerosis Society (ESC/EAS) guidelines for patients with atherosclerotic cardiovascular disease (ASCVD) (< 70 *vs*. < 55 mg/dl, respectively). In the DA VINCI study, residual cardiovascular risk was predicted in ASCVD patients. The extent to which relative and absolute risk might be lowered by achieving ACC/AHA versus ESC/EAS LDL-C recommended approaches was simulated.

**Methods:**

DA VINCI was a cross-sectional observational study of patients prescribed lipid-lowering therapy (LLT) across 18 European countries. Ten-year cardiovascular risk (CVR) was predicted among ASCVD patients receiving stabilized LLT. For patients with LDL-C ≥ 70 mg/dl, the absolute LDL-C reduction required to achieve an LDL-C of < 70 or < 55 mg/dl (LDL-C of 69 or 54 mg/dl, respectively) was calculated. Relative and absolute risk reductions (RRRs and ARRs) were simulated.

**Results:**

Of the 2039 patients, 61% did not achieve LDL-C < 70 mg/dl. For patients with LDL-C ≥ 70 mg/dl, median (interquartile range) baseline LDL-C and 10-year CVR were 93 (81–115) mg/dl and 32% (25–43%), respectively. Median LDL-C reductions of 24 (12–46) and 39 (27–91) mg/dl were needed to achieve an LDL-C of 69 and 54 mg/dl, respectively. Attaining ACC/AHA or ESC/EAS goals resulted in simulated RRRs of 14% (7–25%) and 22% (15–32%), respectively, and ARRs of 4% (2–7%) and 6% (4–9%), respectively.

**Conclusion:**

In ASCVD patients, achieving ESC/EAS LDL-C goals could result in a 2% additional ARR over 10 years versus the ACC/AHA approach.

**Graphical abstract:**

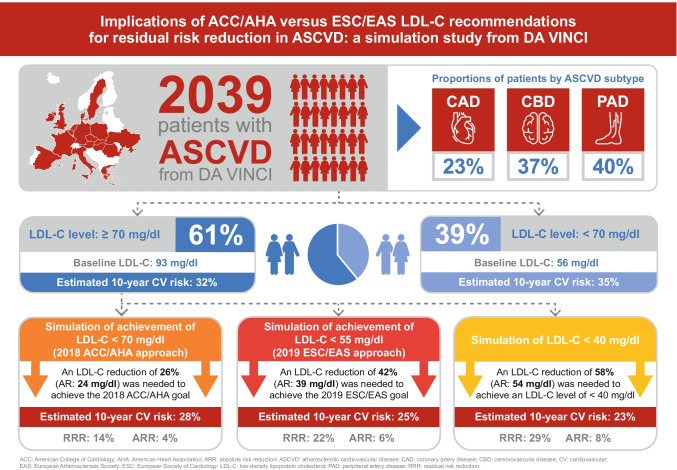

## Introduction

Patients with established atherosclerotic cardiovascular disease (ASCVD) are at the highest risk of cardiovascular events, with high low-density lipoprotein cholesterol (LDL-C) levels being one of the major determinants of the magnitude of their residual risk [1, 2]. As such, these individuals benefit from lipid-lowering therapy (LLT) with statins irrespective of the vascular territory affected (e.g., coronary artery disease [CAD], cerebrovascular disease [CBD], or peripheral artery disease [PAD]) [3–6]. Recent data from large cardiovascular outcomes trials in individuals with ASCVD of add-on LLT (such as proprotein convertase subtilisin/kexin type 9 inhibitors [PCSK9i] and ezetimibe) have demonstrated the benefits of additional lowering of LDL-C levels previously unattainable with statin monotherapy [7–12]. These findings have been incorporated into the current guidelines for patients with ASCVD, with the recommendation that LDL-C should be managed more aggressively with combination LLTs if LDL-C levels are not adequately controlled [13, 14]. However, international guidelines differ with respect to these recommendations, with the 2019 European Society of Cardiology/European Atherosclerosis Society (ESC/EAS) recommendations [13] advocating a more stringent LDL-C goal of < 55 mg/dl in ASCVD patients, compared with their 2016 iteration, which had advocated the less stringent goal of < 70 mg/dl [15]. In contrast, the 2018 American College of Cardiology/American Heart Association (ACC/AHA) guidelines [14] recommend aiming for an LDL-C of < 70 mg/dl, using 70 mg/dl as a threshold to guide additional LLT in ASCVD patients at very-high risk.

The recent EU-Wide Cross-Sectional Observational Study of Lipid-Modifying Therapy Use in Secondary and Primary Care (DA VINCI) [16] provides a valuable opportunity to assess contemporary information regarding how LLTs are used in current practice and their impact on LDL-C levels. Although patients being managed for CAD have been studied extensively in bespoke registries, contemporary data for individuals being managed for CBD or PAD are more limited. Through design, approximately four-fifths of the patients with ASCVD in the DA VINCI study [16] were being managed for CBD or PAD, allowing comparisons across the spectrum of ASCVD phenotypes. The present analysis assesses the use of LLT across these populations, the implications of current practice on the future risk of cardiovascular events, and, through a simulation study, to what extent risk might be mitigated if different guidelines were adopted.

## Methods

### Study Design

The methods and primary results of the DA VINCI study have been described in detail elsewhere [16]. Briefly, DA VINCI was a cross-sectional, observational study of routine clinical management that enrolled 5888 adults (aged ≥ 18 years) in primary and secondary prevention (overall ratio of 1:1) across 18 European countries [16]. Participants must have been prescribed LLT within the 12 months before enrolment and must have had an LDL-C measurement in the 14 months before enrolment. Among secondary prevention participants with established ASCVD, patients being managed for CAD, CBD, or PAD were enrolled in an overall ratio of approximately 1:2:2, respectively. Data were extracted from medical records at a single (enrolment) visit between June 2017 and November 2018. The study protocol (available online at ENCePP; registration no. EUPAS22075 [17]) was approved by the institutional review board or independent ethics committee from each site participating in the study [16].

### Aims and Outcomes

In the present analysis, we assessed LLT use and LDL-C control among patients with established ASCVD, stratified by ASCVD type (by definition, all patients were at very high cardiovascular risk according to 2019 ESC/EAS guidelines [13]). Next, we compared the proportion of patients who were above the LDL-C level of 70 mg/dl recommended by 2018 ACC/AHA guidelines for very high-risk ASCVD patients, versus the attainment of the 2019 ESC/EAS goal of < 55 mg/dl. By means of comparison, we additionally explored, as an extension of the attainment of the 2019 ESC/EAS goal, the proportion of patients achieving LDL-C levels < 40 mg/dl. Finally, we evaluated the implications of current practice and LDL-C goal achievement on the future risk of cardiovascular events using the REduction of Atherothrombosis for Continued Health (REACH) equation [18] and simulation methods. The Cholesterol Treatment Trialists' Collaboration meta-analysis has demonstrated that a 1.0 mmol/l reduction in LDL-C levels leads to a 22% relative risk reduction [4]. For this analysis, we modelled what the anticipated potential added benefit on cardiovascular risk reduction would be if patients not on target were to achieve the recommended LDL-C levels according to 2019 ESC/EAS or 2018 ACC/AHA guidelines.

### Statistical Analysis

Our analysis included all participants with established ASCVD who were receiving stabilized LLT at LDL-C measurement (defined as no change in dose or regimen for at least 28 days prior to LDL-C measurement). The overall ASCVD population was categorized into individuals with LDL-C levels ≥ 70 mg/dl and those with levels < 70 mg/dl. Results are presented for the overall ASCVD group, and further stratified by ASCVD type, namely, CAD, CBD, and PAD (for participants with known atherosclerotic involvement of more than one vascular bed, categorization was made based on the most recent manifestation of vascular disease at enrolment). Results are reported as mean ± standard deviation or median and interquartile range (IQR) for normally and not normally distributed continuous variables, respectively, and as absolute and relative frequencies (n [%]) for categorical variables.

### Calculation of Residual Risk

For each patient, we predicted their (baseline) risk of a subsequent cardiovascular event in the next 10 years (10-year cardiovascular risk) using the REACH equation, based on the individual patient demographics and medical history. The REACH equation predicts the risk of recurrent cardiovascular events and cardiovascular death among outpatients with established ASCVD [18]. The 10-year cardiovascular risk of a subsequent cardiovascular event was calculated by converting the 20-month risk predicted from the REACH equation [18], assuming a constant rate over time (exponential survival function).

For patients with LDL-C levels ≥ 70 mg/dl, we calculated (at an individual level) the absolute and relative reductions in LDL-C required to achieve an LDL-C < 70 mg/dl (2018 ACC/AHA-recommended approach [14]) and LDL-C < 55 mg/dl (2019 ESC/EAS approach), defined for the purposes of this simulation as LDL-C levels of 69 mg/dl and 54 mg/dl, respectively (conservative approach, since the actual LDL-C levels achieved in real life would be expected to be lower if a clinician were to try and lower LDL-C levels to < 70 or < 55 mg/dl, respectively). By means of comparison (as an extension of the attainment of the 2019 ESC/EAS goal), we also explored the reductions in LDL-C required to achieve an LDL-C < 40 mg/dl (defined as 39 mg/dl). In addition, we simulated the relative risk reduction (RRR) by randomly sampling from the inverse probability distribution of the rate ratio per 39 mg/dl from the Cholesterol Treatment Trialists' Collaboration meta-analysis [5]. Finally, we simulated the absolute risk reduction (ARR) and 10-year cardiovascular risk if LDL-C levels of 69, 54, and 39 mg/dl were attained. For comparison, we also assessed the residual risk of cardiovascular events among patients with LDL-C levels < 70 mg/dl.

## Results

### Patient Characteristics

Among participants enrolled in the DA VINCI study, 2039 patients with ASCVD were on stabilized LLT at LDL-C measurement and had data available to allow estimation of risk using the REACH equation; this included 470 (23%), 751 (37%), and 818 (40%) patients being managed for CAD, CBD, and PAD, respectively. Patient characteristics, cardiovascular risk factors, and comorbidities are shown in Table 1, overall and stratified by ASCVD type and by LDL-C < 70 mg/dl or ≥ 70 mg/dl.

**Table 1:** Patient characteristics, cardiovascular risk factors, and comorbidities

	Overall cohort of patients with established ASCVD			Secondary prevention stratified by ASCVD type								
				Coronary artery disease			Cerebrovascular disease			Peripheral artery disease		
	LDL-C < 70 mg/dl	LDL-C ≥ 70 mg/dl	Total	LDL-C < 70 mg/dl	LDL-C ≥ 70 mg /dl	Total	LDL-C < 70 mg/dl	LDL-C ≥ 70 mg/dl	Total	LDL-C < 70 mg/dl	LDL-C ≥ 70 mg/dl	Total
*n*	801	1238	2039	207	263	470	268	483	751	326	492	818
Female sex, *n* (%)	200 (25.0)	458 (37.0)	658 (32.3)	42 (20.3)	70 (26.6)	112 (23.8)	77 (28.7)	213 (44.1)	290 (38.6)	77 (28.7)	175 (35.6)	256 (31.3)
Race, white, *n* (%)	721 (90.0)	1160 (93.7)	1881 (92.3)	180 (87.0)	246 (93.5)	426 (90.6)	243 (90.7)	446 (92.3)	689 (91.7)	298 (91.4)	468 (95.1)	766 (93.6)
Age, years, mean (SD)	68.7 (9.8)	68.0 (10.0)	68.3 (9.9)	67.4 (9.7)	67.3 (10.2)	67.4 (9.9)	68.7 (10.6)	67.9 (10.3)	68.2 (10.4)	69.6 (9.1)	68.4 (9.5)	68.9 (9.4)
Systolic blood pressure, mmHg, mean (SD)	134.8 (18.3)	135.4 (17.2)	135.2 (17.6)	133.2 (17.9)	135.3 (16.5)	134.4 (17.1)	133.8 (15.2)	135.2 (17.1)	134.7 (16.5)	136.6 (20.7)	135.7 (17.7)	136.1 (18.9)
Diastolic blood pressure, mmHg, mean (SD)	76.0 (10.9)	77.4 (10.8)	76.9 (10.8)	77.4 (10.6)	77.9 (11.1)	77.7 (10.9)	77.3 (10.5)	78.4 (10.6)	78.0 (10.6)	74.1 (11.1)	76.1 (10.6)	75.3 (10.8)
Body mass index, kg/m^2^, median (Q1, Q3)	28.3 (25.2, 31.4)	27.4 (24.9, 30.9)	27.8 (25.0, 31.1)	28.4 (25.8, 30.9)	27.7 (25.3, 31.3)	28.0 (25.4, 31.1)	28.7 (25.7, 32.1)	27.6 (25.0, 30.9)	27.9 (25.2, 31.4)	27.9 (24.3, 31.2)	27.1 (24.7, 30.8)	27.5 (24.6, 31.0)
Smoking history, *n* (%)												
Non-smoker	288 (36.0)	504 (40.7)	792 (38.8)	95 (45.9)	123 (46.8)	218 (46.4)	124 (46.3)	265 (54.9)	389 (51.8)	69 (21.2)	116 (23.6)	185 (22.6)
Ex-smoker	372 (46.4)	503 (40.6)	875 (42.9)	92 (44.4)	112 (42.6)	204 (43.4)	103 (38.4)	154 (31.9)	257 (34.2)	177 (54.3)	237 (48.2)	414 (50.6)
Light smoker	50 (6.2)	71 (5.7)	121 (5.9)	8 (3.9)	12 (4.6)	20 (4.3)	18 (6.7)	23 (4.8)	41 (5.5)	24 (7.4)	36 (7.3)	60 (7.3)
Moderate smoker	56 (7.0)	94 (7.6)	150 (7.4)	6 (2.9)	9 (3.4)	15 (3.2)	13 (4.9)	26 (5.4)	39 (5.2)	37 (11.3)	59 (12.0)	96 (11.7)
Heavy smoker	32 (4.0)	63 (5.1)	95 (4.7)	6 (2.9)	6 (2.3)	12 (2.6)	7 (2.6)	13 (2.7)	20 (2.7)	19 (5.8)	44 (8.9)	63 (7.7)
Missing	3 (0.4)	3 (0.2)	6 (0.3)	0 (0.0)	1 (0.4)	1 (0.2)	3 (1.1)	2 (0.4)	5 (0.7)	0 (0.0)	0 (0.0)	0 (0.0)
Vascular beds involved,^a^ *n* (%)												
Coronary	342 (42.7)	427 (34.5)	769 (37.7)	206 (99.5)	260 (98.9)	466 (99.1)	29 (10.8)	49 (10.1)	78 (10.4)	107 (32.8)	118 (24.0)	225 (27.5)
Cerebrovascular	318 (39.7)	548 (44.3)	866 (42.5)	11 (5.3)	12 (4.6)	23 (4.9)	267 (99.6)	476 (98.6)	743 (98.9)	40 (12.3)	60 (12.2)	100 (12.2)
Peripheral	338 (42.2)	509 (41.1)	847 (41.5)	13 (6.3)	9 (3.4)	22 (4.7)	11 (4.1)	14 (2.9)	25 (3.3)	314 (96.3)	486 (98.8)	800 (97.8)
Diabetes mellitus, *n *(%)	406 (50.7)	487 (39.3)	893 (43.8)	104 (50.2)	96 (36.5)	200 (42.6)	129 (48.1)	170 (35.2)	299 (39.8)	173 (53.1)	221 (44.9)	394 (48.2)
Hypertension, *n *(%)	629 (78.5)	971 (78.4)	1600 (78.5)	158 (76.3)	198 (75.3)	356 (75.7)	199 (74.3)	393 (81.4)	592 (78.8)	272 (83.4)	380 (77.2)	652 (79.7)
Chronic kidney disease ≥ Grade 3, *n* (%)	99 (11.0)	118 (9.5)	206 (10.1)	16 (7.7)	22 (8.4)	38 (8.1)	23 (8.6)	36 (7.5)	59 (7.9)	49 (15.0)	60 (12.2)	109 (13.3)

Patients were categorized as coronary, cerebrovascular, or peripheral as mutually exclusive categories on the date of visit, whereas vascular bed involvement was captured on the “Cardiovascular event history entry” page and a patient could have more than one event recorded. ^a^Data do not sum to 100% for each ASCVD subtype because the data were collected separately in the case report form. *ASCVD* atherosclerotic cardiovascular disease; *LDL-C* low-density lipoprotein cholesterol; *SD* standard deviation

### LLT Use and LDL-C Levels in Patients with ASCVD

LLT use is shown in Table 2 and consisted mostly of statin monotherapy. Use of moderate-intensity statins as monotherapy ranged from 35% among patients with CAD to 47% in patients with CBD. High-intensity statin monotherapy was used in 44% of patients with CAD and 36% of patients with either CBD or PAD. Ezetimibe was used in combination with statins in 15% of patients with CAD, 7% of patients with CBD, and 8% of patients with PAD. In each group, PCSK9i was used in combination with statins and/or ezetimibe in less than 2% of patients.

**Table 2:** LLT use at LDL-C measurement

	Overall cohort of patients with established ASCVD			Secondary prevention stratified by ASCVD type								
				Coronary artery disease			Cerebrovascular disease			Peripheral artery disease		
	LDL-C < 70 mg/dl	LDL-C ≥ 70 mg/dl	Total	LDL-C < 70 mg/dl	LDL-C ≥ 70 mg/dl	Total	LDL-C < 70 mg/dl	LDL-C ≥ 70 mg/dl	Total	LDL-C < 70 mg/dl	LDL-C ≥ 70 mg/dl	Total
*n*	801	1238	2039	207	263	470	268	483	751	326	492	818
Any statin	781 (97.5)	1131 (91.4)	1912 (93.8)	204 (98.6)	251 (95.4)	455 (96.8)	264 (98.5)	435 (90.1)	699 (93.1)	313 (96.0)	445 (90.4)	758 (92.7)
Low-intensity statin^a^	14 (1.7)	43 (3.5)	57 (2.8)	5 (2.4)	7 (2.7)	12 (2.6)	4 (1.5)	19 (3.9)	23 (3.1)	5 (1.5)	17 (3.5)	22 (2.7)
Moderate-intensity statin^b^	346 (43.2)	606 (48.9)	952 (46.7)	75 (36.2)	115 (43.7)	190 (40.4)	120 (44.8)	246 (50.9)	366 (48.7)	151 (46.3)	245 (49.8)	396 (48.4)
High-intensity statin^c^	397 (49.6)	461 (37.2)	858 (42.1)	120 (58.0)	120 (45.6)	240 (51.1)	135 (50.4)	163 (33.7)	298 (39.7)	142 (43.6)	178 (36.2)	320 (39.1)
Unknown-intensity statin^d^	24 (3.0)	21 (1.7)	45 (2.2)	4 (1.9)	9 (3.4)	13 (2.8)	5 (1.9)	7 (1.4)	12 (1.6)	15 (4.6)	5 (1.0)	20 (2.4)
Ezetimibe^e^	124 (15.5)	118 (9.5)	242 (11.9)	38 (18.4)	43 (16.3)	81 (17.2)	31 (11.6)	38 (7.9)	69 (9.2)	55 (16.9)	37 (7.5)	92 (11.2)
PCSK9i^f^	18 (2.2)	12 (1.0)	30 (1.5)	3 (1.4)	2 (0.8)	5 (1.1)	6 (2.2)	4 (0.8)	10 (1.3)	9 (2.8)	6 (1.2)	15 (1.8)
*Combination LLT at LDL-C measurement*												
Low-intensity statin monotherapy	9 (1.1)	38 (3.1)	47 (2.3)	3 (1.4)	6 (2.3)	9 (1.9)	3 (1.1)	16 (3.3)	19 (2.5)	3 (0.9)	16 (3.3)	19 (2.3)
Moderate-intensity statin monotherapy	311 (38.8)	576 (46.5)	887 (43.5)	61 (29.5)	103 (39.2)	164 (34.9)	113 (42.2)	238 (49.3)	351 (46.7)	137 (42.0)	235 (47.8)	372 (45.5)
High-intensity statin monotherapy	344 (42.9)	420 (33.9)	764 (37.5)	102 (49.3)	103 (39.2)	205 (43.6)	116 (43.3)	151 (31.3)	267 (35.6)	126 (38.7)	166 (33.7)	292 (35.7)
Ezetimibe combo^g^	102 (12.7)	87 (7.0)	189 (9.3)	34 (16.4)	38 (14.4)	72 (15.3)	26 (9.7)	24 (5.0)	50 (6.7)	42 (12.9)	25 (5.1)	67 (8.2)
PCSK9i combo^h^	16 (2.0)	8 (0.6)	24 (1.2)	2 (1.0)	0 (0.0)	2 (0.4)	6 (2.2)	3 (0.6)	9 (1.2)	8 (2.5)	5 (1.0)	13 (1.6)
Other LLT^i^	19 (2.4)	109 (8.8)	128 (6.3)	5 (2.4)	13 (4.9)	18 (3.8)	4 (1.5)	51 (10.6)	55 (7.3)	10 (3.1)	45 (9.1)	55 (6.7)

Data are *n* (%) of patients. ^a^Patients treated with low-intensity statin (with or without other LLT); ^b^Patients treated with moderate-intensity statin (with or without other LLT); ^c^Patients treated with high-intensity statin (with or without other LLT); ^d^Patients receiving statin treatment of unknown intensity (with or without other LLT); ^e^Patients treated with ezetimibe (with or without other LLT); ^f^Patients treated with PCSK9i, alirocumab, or evolocumab; ^g^Patients treated with ezetimibe plus statin of moderate/high/unknown intensity; ^h^Patients treated with PCSK9i plus statin of low/moderate/high/unknown intensity, or PCSK9i plus ezetimibe, or PCSK9i plus statin and ezetimibe; ^i^Patients treated with ezetimibe without statin or PCSK9i, PCSK9i without statin or ezetimibe, ezetimibe plus statin of low/unknown intensity without ezetimibe or PCSK9i, other LLTs such as fibrates, and fish oils. *ASCVD* atherosclerotic cardiovascular disease; *combo* combination; *LDL-C* low-density lipoprotein cholesterol; *LLT* lipid-lowering therapy; *PCSK9i* proprotein convertase subtilisin/kexin type 9 inhibitor

**Fig. 1:**
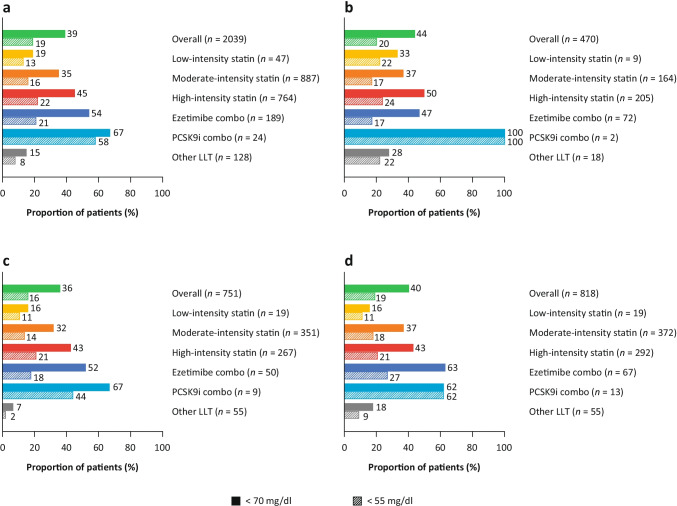
Achievement of LDL-C levels < 70 mg/dl and < 55 mg/dl in (**a**) the overall cohort of patients with established ASCVD and stratified by type of ASCVD, namely, (**b**) coronary artery disease, (**c)** cerebrovascular disease, and (**d)** peripheral artery disease. The proportion of individuals within each ASCVD group who achieved LDL-C levels of < 70 mg/dl and < 55 mg/dl are described. Overall, 39% of patients achieved an LDL-C level < 70 mg/dl and 19% of patients achieved an LDL-C level < 55 mg/dl. The achievement of an LDL-C level < 70 mg/dl was more likely in patients receiving combination therapy with either ezetimibe or a PCSK9i than patients on statin monotherapy. ASCVD, atherosclerotic cardiovascular disease; LDL-C, low-density lipoprotein cholesterol; LLT, lipid-lowering therapy; PCSK9i, proprotein convertase subtilisin/kexin type 9 inhibitor

The proportion of individuals, overall and within each ASCVD group, who achieved LDL-C levels of < 70 mg/dl and < 55 mg/dl are shown in Fig. 1. Overall, 39% of patients achieved an LDL-C level < 70 mg/dl and 19% achieved an LDL-C level < 55 mg/dl (Fig. 1a). The achievement of an LDL-C level < 70 mg/dl was more likely in patients receiving combination therapy with either ezetimibe or a PCSK9i than in patients on statin monotherapy. Although used infrequently, 58% of patients receiving PCSK9i in combination with other LLTs attained an LDL-C level < 55 mg/dl. The proportion of patients across ASCVD subtypes with LDL-C levels < 70 mg/dl was 44%, 36%, and 40% for the CAD, CBD, and PAD groups, respectively, and 20%, 16%, and 19% for LDL-C levels < 55 mg/dl (Fig. 1b–d).

### Residual Risk Among Those with LDL-C ≥ 70 mg/dl and < 70 mg/dl

**Fig. 2:**
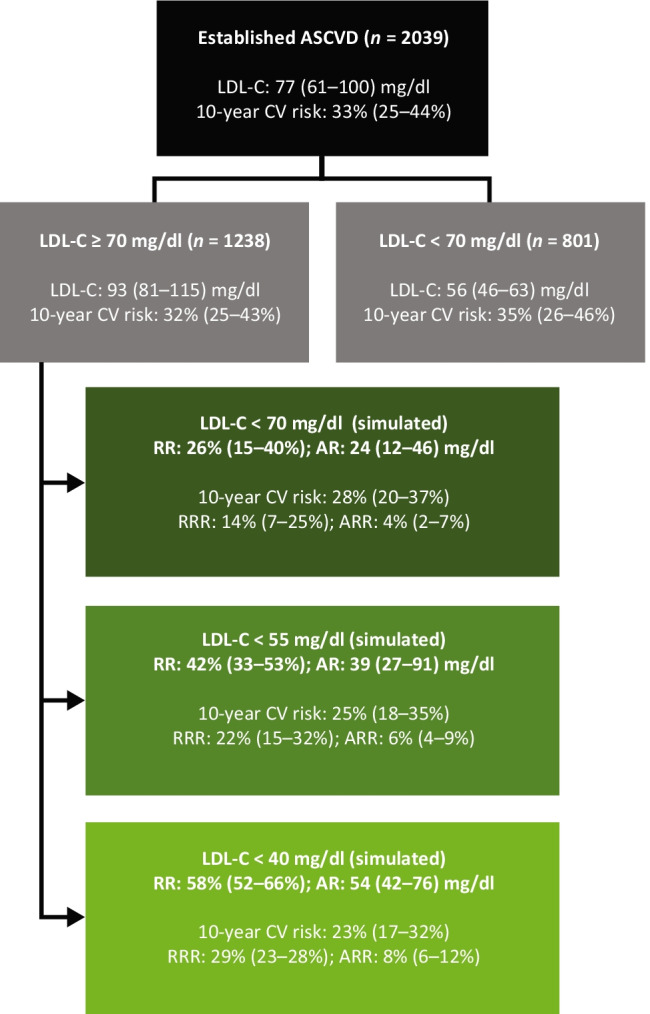
Simulated risk reduction in patients with established ASCVD. Simulated risk reductions associated with attainment of 2019 ESC/EAS LDL-C goal of < 55 mg/dl and 2018 American College of Cardiology/American Heart Association (ACC/AHA) recommended approach of LDL-C < 70 mg/dl, respectively. Ten-year CV risk was lower with attainment of 2019 ESC/EAS goals (25%) versus the 2018 ACC/AHA approach (28%). LDL-C reductions of 24 (12–46) and 39 (27–91) mg/dl were needed to achieve LDL-C of 69 and 54 mg/dl, respectively. AR, absolute reduction; ASCVD, atherosclerotic cardiovascular disease; ARR, absolute risk reduction; CV, cardiovascular; EAS, European Atherosclerosis Society; ESC, European Society of Cardiology; LDL-C, low-density lipoprotein cholesterol; RR, risk reduction; RRR, relative risk reduction

Overall, 61% (1238/2039) of patients had an LDL-C level ≥ 70 mg/dl (Fig. 2), with a median (IQR) LDL-C level of 93 (81–115) mg/dl. The median (IQR) predicted 10-year cardiovascular risk for these patients was 32% (25–43%). Among patients with LDL-C < 70 mg/dl (39% [801/2039]), median (IQR) LDL-C levels were 56 (46–63) mg/dl. Among these patients, the median (IQR) predicted 10-year cardiovascular risk was 35% (26–46%). Demographic characteristics varied between those who had LDL-C levels ≥ 70 mg/dl or < 70 mg/dl, with a slightly higher prevalence of risk factors such as a history of smoking and diabetes in the group with levels < 70 mg/dl (Table 1). The proportion of participants with diabetes was higher in patients with LDL-C < 70 mg/dl (51% of patients with a median LDL-C of 56 [46–63] mg/dl) compared with those with LDL-C levels ≥ 70 mg/dl (39% with a median LDL-C of 93 [81–115] mg/dl) (Table 1).

### Simulated Risk Reduction on Achieving an LDL-C < 70 mg/dl

The cohort of patients with LDL-C levels ≥ 70 mg/dl required a median (IQR) 26% (15–40%) reduction (absolute reduction of 24 [12–46] mg/dl) to lower their LDL-C levels to 69 mg/dl (Fig. 2). The median (IQR) simulated RRR and ARR of cardiovascular events were 14% (7–25%) and 4% (2–7%), respectively. The median (IQR) simulated residual 10-year cardiovascular risk was 28% (20–37%).

**Fig. 3:**
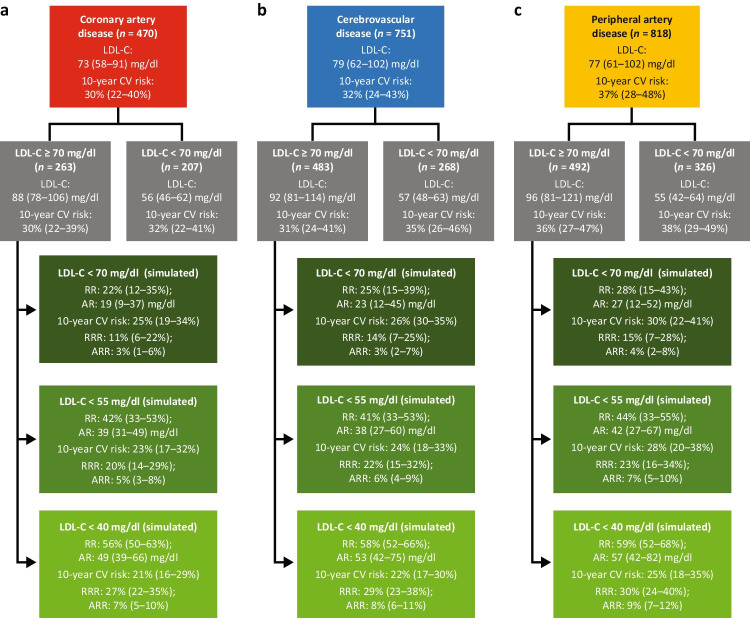
Simulated risk reduction in the (**a**) coronary artery disease, (**b**) cerebrovascular disease, and (**c)** peripheral artery disease groups. Simulated risk reductions associated with attainment of 2019 ESC/EAS (< 55 mg/dl) and 2018 ACC/AHA (< 70 mg/dl) LDL-C recommendations stratified by ASCVD subtype. Patients being managed for coronary artery disease, cerebrovascular disease, and peripheral artery disease each had similar 10-year CV risk estimates. ACC American College of Cardiology; AHA, American Heart Association; AR, absolute reduction; ARR, absolute risk reduction; CV, cardiovascular; EAS, European Atherosclerosis Society; ESC, European Society of Cardiology; LDL-C, low-density lipoprotein cholesterol; RR, risk reduction; RRR, relative risk reduction

In the CAD group, patients with LDL-C levels ≥ 70 mg/dl required a median (IQR) 22% (12–35%) reduction (absolute reduction of 19 [9–37] mg/dl) to lower their LDL-C levels to 69 mg/dl (Fig. 3a). The median (IQR) simulated RRR and ARR of cardiovascular events were 11% (6–22%) and 3% (1–6%), respectively. For patients with CAD, attaining an LDL-C level < 70 mg/dl would result in a lower median (IQR) simulated residual 10-year cardiovascular risk of 25% (19–34%).

In the CBD group, patients with LDL-C levels ≥ 70 mg/dl required a median (IQR) 25% (15–39%) reduction to lower their LDL-C levels to 69 mg/dl, with a median (IQR) absolute reduction of 23 (12–45) mg/dl (Fig. 3b). The median (IQR) simulated RRR and ARR of cardiovascular events were 14% (7–25%) and 3% (2–7%), respectively. The median (IQR) simulated residual 10-year cardiovascular risk was 26% (30–35%).

In the PAD group, patients with LDL-C levels ≥ 70 mg/dl required a median (IQR) 28% (15–43%) reduction to lower their LDL-C levels to 69 mg/dl, with a median (IQR) absolute reduction of 27 (12–52) mg/dl (Fig. 3c). The median (IQR) simulated RRR and ARR of cardiovascular events were 15% (7–28%) and 4% (2–8%), respectively. The median (IQR) simulated residual 10-year cardiovascular risk was 30% (22–41%).

### Simulated Risk Reduction on Achieving an LDL-C < 55 mg/dl

In order for patients with LDL-C levels ≥ 70 mg/dl to reach levels of 54 mg/dl, a median (IQR) 42% (33–53%) reduction in LDL-C was required (absolute reduction of 39 [27–91] mg/dl) (Fig. 2). The median (IQR) simulated RRR and ARR of cardiovascular events were 22% (15–32%) and 6% (4–9%), respectively. The median (IQR) simulated residual 10-year cardiovascular risk was 25% (18–35%).

In the CAD group, patients with LDL-C levels ≥ 70 mg/dl required a median (IQR) 42% (33–53%) reduction (absolute reduction of 39 [31–49] mg/dl) to lower their LDL-C levels to 54 mg/dl (Fig. 3). The median (IQR) simulated RRR and ARR of cardiovascular events were 20% (14–29%) and 5% (3–8%), respectively. The resulting median (IQR) simulated residual 10-year cardiovascular risk was 23% (17–32%).

In the CBD group, those with LDL-C ≥ 70 mg/dl required a median (IQR) 41% (33–53%) reduction (absolute reduction of 38 [27–60] mg/dl) to lower their LDL-C levels to 54 mg/dl (Fig. 3b). The median (IQR) simulated RRR and ARR of cardiovascular events were 22% (15–32%) and 6% (4–9%), respectively. The resulting median (IQR) simulated residual 10-year cardiovascular risk was 24% (18–33%).

In the PAD group, patients with LDL-C levels ≥ 70 mg/dl required a median (IQR) 44% (33–55%) reduction (absolute reduction of 42 [27–67] mg/dl) to lower their LDL-C levels to 54 mg/dl (Fig. 3c). The median (IQR) simulated RRR and ARR of cardiovascular events were 23% (16–34%) and 7% (5–10%), respectively. The resulting median (IQR) simulated residual 10-year cardiovascular risk was 28% (20–38%).

### Simulated Risk Reduction on Achieving an LDL-C < 40 mg/dl

In order for patients with LDL-C levels ≥ 70 mg/dl to reach levels of 39 mg/dl, a median (IQR) 58% (52–66%) reduction in LDL-C was required (absolute reduction of 54 [42–76] mg/dl) (Fig. 2). The median (IQR) simulated RRR and ARR of cardiovascular events were 29% (23–28%) and 8% (6–12%), respectively. The resulting median (IQR) simulated residual 10-year cardiovascular risk was 23% (17–32%). The corresponding values for each subgroup are presented in Fig. 3.

## Discussion

The DA VINCI study, conducted across 18 countries and 128 sites in Europe, provided an opportunity to assess the patterns of LLT use in contemporary practice, as well as gaps between guidelines and their implementation in patients with manifestations of ASCVD. Of the 2039 patients with ASCVD in this study, the majority (61%) did not achieve an LDL-C goal of < 70 mg/dl and only approximately one-fifth of patients (19%) reached the more stringent goal of < 55 mg/dl. Simulations from this study indicate that median LDL-C reductions of 24 (12–46) and 39 (27–61) mg/dl would be needed in this population to achieve an LDL-C level of 69 and 54 mg/dl, respectively. If LDL-C levels of 54 mg/dl were achieved, the estimated ARR would be expected to be 6% over 10 years as compared with an estimated ARR of 4% if LDL-C levels of 69 mg/dl were achieved. These results have implications for current clinical practice and highlight the extent that current residual risk among ASCVD patients receiving statins might be mitigated if the 2019 ESC/EAS guidelines were achieved in a population with ASCVD similar to the DA VINCI cohort.

The DA VINCI study shows that, in patients with ASCVD, LLT use consisted of monotherapy-based treatments in approximately 80–85% of cases. Use of combination therapy (e.g., statins with ezetimibe and/or PCSK9i) was low (< 20%), but use of combination therapy was twice as frequent among patients being managed for CAD than among those with CBD or PAD. The result of this monotherapy-based approach is that, among patients with major manifestations of ASCVD, the majority failed to attain either the 2019 ESC/EAS or the 2018 ACC/AHA LDL-C recommendations with current LLT use. In addition, among patients not achieving the 2018 ACC/AHA LDL-C recommendation of < 70 mg/dl), mean LDL-C levels were approximately 93 mg/dl (or 24 mg/dl higher than the recommended figure), with an estimated 10-year risk of cardiovascular events of 32%. Notably, even among patients who had achieved an LDL-C < 70 mg/dl (with a mean LDL-C of 56 mg/dl), the estimated 10-year risk of cardiovascular events was 35%.

The higher risk observed in these patients is likely due to additional comorbidities such as diabetes or a history of smoking. This observation highlights the importance of absolute risk when making clinical decisions about optimal LDL-C control for individual patients [19].

Large-scale Mendelian randomization genetic studies that simulate LLT use suggest that the cardiovascular benefits of LDL-C lowering should be similar, irrespective of the mechanism by which lowering is achieved when standardized for the same absolute difference in LDL-C [20, 21]. Furthermore, comparisons of pharmacological approaches to LDL-C lowering suggest that conflicting findings from trials can be harmonized when the observed RRR in cardiovascular events is standardized per an approximately 39 mg/dl (1 mmol/l) lowering in LDL-C and the number of years of treatment [20, 22]. Current guidelines recommend a stepwise approach strategy [13,14]. This strategy is based on the iterative development of evidence from randomized trials and economic considerations, with statins being the cornerstone of LDL-C lowering and subsequent therapies being added on. An impact of this approach is that, in routine clinical practice, there is an inevitable delay in the use of evidence-based therapies. For instance, although ezetimibe is widely available as a generic therapy, it remains underutilized. In the present study, the enrolment centres with participating physicians with a primary interest in lipid management only used ezetimibe in combination with statins in 9% of patients. This proportion is even lower when ezetimibe use is measured in unselected cohorts available through assessment of electronic health records [23].

There are notable differences between the 2019 ESC/EAS and 2018 ACC/AHA guidelines in their clinical approach to LLT. The 2019 ESC/EAS guidelines recommend that all patients with ASCVD are classified as very high risk and that an LDL-C goal of < 55 mg/dl for these patients is to be achieved. In contrast, the 2018 ACC/AHA guidelines classify high and very high-risk patients by the presence or absence of additional very high-risk characteristics and recommend an LDL-C ≥ 70 mg/dl to guide intensification of therapy among those at very high risk. The 2016 ESC/EAS guidelines [15] were closer to the current 2018 ACC/AHA guidelines in the sense of recommending an LDL-C goal of < 70 mg/dl for patients with ASCVD.

In the DA VINCI study, full implementation of the 2016 ESC/EAS or 2018 ACC/AHA guidelines would mean that an average LDL-C reduction of 24 mg/dl (relative reduction of 26%) would be required for patients not achieving an LDL-C < 70 mg/dl. It has been reported that doubling statin dosing produces approximately a further 6% reduction in LDL-C [24] and the addition of ezetimibe results in up to 25% further lowering [25]. As the IQR for absolute reductions in LDL-C ranged from 12 to 46 mg/dl, the majority of patients might achieve LDL-C < 70 mg/dl if moderate-intensity statins were optimized to high-intensity statins and ezetimibe was prescribed more frequently, with a smaller proportion of patients requiring PCSK9i. If LDL-C levels of 69 mg/dl were achieved, the estimated ARR would be expected to be 4%, with a simulated 10-year risk of 28% after attainment of that LDL-C level. These data are consistent with simulations conducted using US claims data [26]. In contrast, achieving the 2019 ESC/EAS goal of < 55 mg/dl would require greater absolute reductions of 39 mg/dl (IQR 27–91 mg/dl), with a relative reduction of 42%, to achieve an LDL-C level of 54 mg/dl. As a result, an increased use of PCSK9i with higher-intensity statins would likely be required to reach lower LDL-C goals. Inevitably, there may be some patients who may not be able to tolerate higher intensity regimens, and intensive statin therapy has been previously shown to be associated with an increased risk of developing new-onset diabetes with approximately one additional case per 1000 patients per year. However, this risk was outweighed by three predicted cardiovascular events being prevented during the same time period [27]. It is worth noting that from the HEYMANS real-world registry in Europe, when evolocumab treatment is used as part of combination therapy with an oral LLT such as statins and/or ezetimibe, this combination resulted in more patients attaining LDL-C levels of < 55 mg/dl in comparison with PCSK9i monotherapy, further reinforcing the importance of combination therapies [28]. Future DA VINCI analyses will examine the simulated LDL-C reductions associated with following specific treatment intensification pathways (e.g., treatment with high-intensity statins alone, adding ezetimibe, or adding ezetimibe and PCSK9i). Our observations are consistent with recent findings from the SWEDEHEART Registry, in which a population of patients who experienced a recent post-myocardial infarction (MI) was studied [29]. If LDL-C levels of 54 mg/dl were achieved, the estimated ARR would be expected to be 6%, with a simulated 10-year cardiovascular risk of 25%. It should be noted that, while our simulations were based on the conservative approach of patients achieving LDL-C levels of 69 mg/dl and 54 mg/dl, the actual LDL-C levels that would be achieved in clinical practice would likely be lower (and the associated benefits higher) if clinicians were to reduce LDL-C levels to < 70 mg/dl or < 55 mg/dl, particularly if optimized combination LLT was used.

Overall, patients being managed for CAD, CBD, and PAD each had similar 10-year cardiovascular risk estimates when receiving mainly monotherapy regimens, and baseline characteristics were largely similar between these groups. Historically, populations with CBD and PAD are understudied in large registries that usually focus on patients with MI and CAD. In patients with CAD, the benefits of LDL-C lowering with the use of ezetimibe and PCSK9i have been demonstrated in clinical trials [9–11]. As most trials recruited patients on the basis of CAD, and thus few patients have CBD or PAD only, it is possible that this has contributed to delays in evidence-based LLT use in these patient groups. Nevertheless, a recent study in patients with stroke showed that lower LDL-C levels achieved through more intensive LLT regimens (including ezetimibe alone, or with statin combination therapies) resulted in reductions in cardiovascular events [30].

The current 2018 ACC/AHA guidance to aid intensification of LLT is largely qualitative; the guidance identifies multiple different univariate very high-risk conditions [11, 31] but does not allow quantitative estimation of cardiovascular risk and thus does not allow absolute benefit estimation and re-estimation of risk after further LDL-C lowering. In this regard, our simulation study does not allow direct comparisons between the implementation of the 2018 ACC/AHA and 2019 ESC/EAS guidelines. Nevertheless, our study provides information that, at a population level, adoption of the lower LDL-C goal for patients with ASCVD as recommended by the 2019 ESC/EAS guidelines is expected to result in greater ARR and lower residual risk once the more stringent goals have been achieved.

Another interesting observation from our study is that, using quantitative approaches to estimate 10-year cardiovascular risk, the estimated 10-year cardiovascular risk was 35% in patients who achieved an LDL-C < 70 mg/dl (2018 ACC/AHA approach) with an average LDL-C of 56 mg/dl. These patients were more likely to have additional comorbidities such as diabetes or a history of smoking but also had increased use of more potent LLT regimens, including combination therapy. This may suggest that physicians are already identifying certain very high-risk comorbidity or lifestyle factors and treating such patients more aggressively. However, our observations suggest that achieving LDL-C goals without due consideration to absolute risk is only partly informative and may result in potentially modifiable risks being unaddressed [19]. A practical implication of this simulation is that the relative benefit from LLT is proportional to absolute reduction in LDL-C. A patient who is receiving LLT with an LDL-C level > 55 mg/dl would require more potent add-on therapies such as PCSK9i, which reduce LDL-C by 50–60%, thus likely achieving a further 28–34 mg/dl reduction in LDL-C levels. This is in contrast to simply ensuring that all patients achieve an LDL-C of 39 mg/dl, which could be achieved with additional oral therapies but would result on average in only a further 17 mg/dl lowering of LDL-C levels in that population. Both approaches have merits from a population health standpoint in terms of cardiovascular events prevented, but there are implications for drug acquisition costs, which also need to be considered.

The strengths and limitations of the present study merit consideration. Although a systematic approach was used to assess patients, the participating sites were likely to have focused on lipid management and prevention of cardiovascular events, so the findings may represent a better than average management scenario. The use of LLT may vary across countries, reflecting differences in healthcare systems, prevalence of other major cardiovascular risk factors, drug acquisition costs, and local guidelines, and may not necessarily reflect practice in other regions. Despite this, goal attainment is generally determined by the starting level of LDL-C prior to treatment, which would not be expected to vary significantly across regions. Furthermore, the LLT regimens used may vary across countries, but as the effects of LLT on percentage reduction in LDL-C should not vary across the same populations, the findings are meaningful to other populations. We used a simulation approach to provide inferences about potential treatment benefits rather than using observational longitudinal data. These simulations were based on the REACH equation, which has been derived from a large, global cohort [18], albeit with a relatively short follow-up (20 months). While we acknowledge that other risk equations are available, REACH is well established for examining risk in ASCVD patients [32]. We approximated the predictions from the REACH equation to 10-year cardiovascular risk, which could under- or overestimate risk. This under- or overestimation would apply equally to all individuals included, and hence, our study results could be considered as illustrative, exploring the concepts of different LDL-C goals within the caveats of the simulation and population studied. It is worth noting that the DA VINCI cohort described in this study included both very high-risk and high-risk ASCVD patients according to the current ACC/AHA criteria [14]. Hence, the present simulation may have underestimated cardiovascular risk and therefore absolute risk reduction among very high-risk patients (according to the ACC/AHA criteria), as the REACH equation was derived from a cohort that included patients who would not be considered as very high risk [33–35]. However, baseline risk varies across populations, whether one uses ESC/EAS or ACC/AHA criteria for very high risk, and these categories are qualitative rather than quantitative in providing measures of absolute risk and benefit. Therefore, the present study should be considered as illustrative within the limitations described. Finally, as with many studies, an additional limitation of our study was the homogeneous ethnicity of the DA VINCI cohort, in which over 90% of participants were white [33–35].

## Conclusions

In a multi-country broad cohort of patients with ASCVD, the majority of patients who were mostly treated with statin monotherapy did not achieve 2019 ESC/EAS or 2018 ACC/AHA LDL-C recommended goals. Implementation of these guidelines would require greater use of combination therapies, with the more stringent 2019 ESC/EAS goal expected to yield better health outcomes than the 2018 ACC/AHA LDL-C approach.

## Data Availability

Qualified researchers may request data from Amgen clinical studies. Complete data are available at the following: https://wwwext.amgen.com/science/clinical-trials/clinical-data-transparency-practices/
